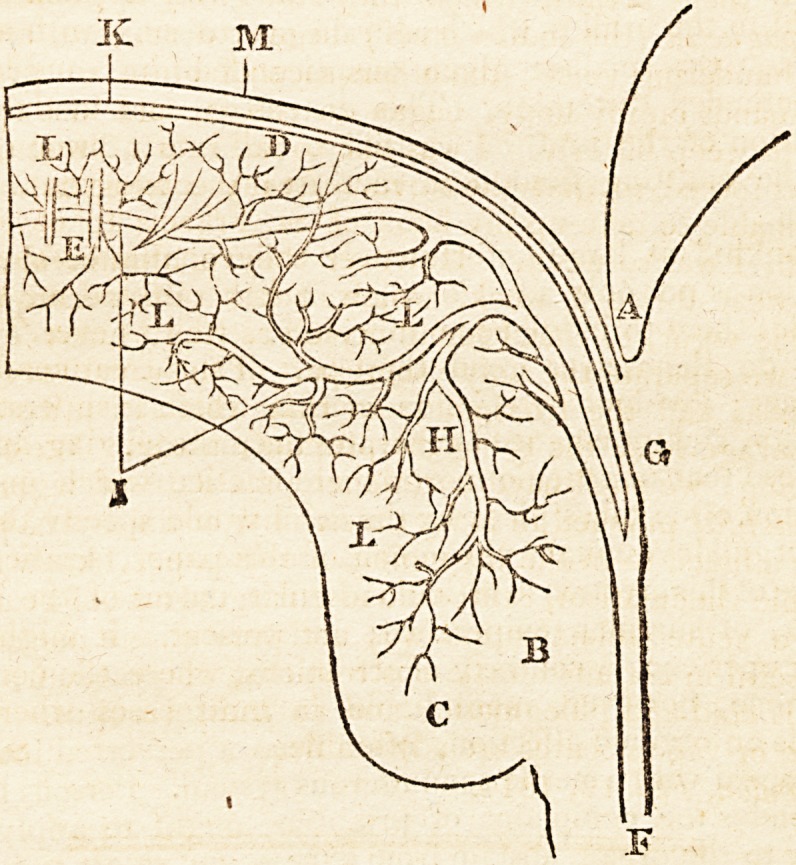# Observations on Peculiar Diseases Incident to the Sexes of the Human Species

**Published:** 1814-03

**Authors:** Alex. Ramsay

**Affiliations:** No. 3, Villa Place, Walworth Common, London


*London, Feb.* 12, 1814.

---

*For the Medical and Physical Journal.*

### *Observations on peculiar Diseases incident to the Sexes of the Human Species; by* Alex. Ramsay, M.D.

### THE MALE SEX.

MANY years since, when I gave lectures on the physiology of the human organs, in Surgeon's-square, in Edinburgh, I illustrated the error of authors, in assigning the erection of the penis to the compression of the vena ipsius ipsius penis, by its approximating the os pubis. I grant that erection causes compression; but what gave rise to erection? Compression is, an effect of a preceding cause. Since the period I mention, the ingenious Messrs. Bell and Fyfe have given descriptions analogous to the following demonstration, in their systems of anatomy; but the ancient doctrines were universally detailed at the time I gave my first lectures on the subject. The inductions, however, depending on the structure demonstrated in the annexed diagram; become the object of the following observations. Priapism, a malady so very universal, teasing, and mistaken, seems explicable from the phenomena of the diseased state of the arteries distributed in the body of the penis.

A, is the os pubis; B, the crus penis; C, the bulb of the urethra; D, the external, dense, and elastic substance which composes the outer surface of the body of the penis; E, the frenula which regulates the distension; F, the root of the arteria penis; G, ramus of the dorsum, continued to the glans; H, ramus of the urethra, spread upon the cells; I, rami of the body, diffused upon the cells of the body of the penis. The ramus G seems the proper arteria nutritia of the organ; its rami anastomose with each other and the

 veins.

veins. The rami H, I, seem the erecting or distending causes; they do not anastomose, but possess absolute open mouths, which effuse the arterial blood into the cells: thus erection is effected, and the compression of the vena ipsius penis is merely a subsequent consequence; we can effect this by injection of the artery.*

If I am correct in the notion of the entire structure of this organ, the phenomena of erection seem the following: the external, dense, elastic substance K, presents three phenomena; 1st, it is susceptible of a physical collapsed state when unexcited; 2dly, it is susceptible of being distended when forced by the effusion of the arteries into the cells L; 3dly, its elasticity occasions its return to the physical collapse when the orgasm has ceased, thus forcing back the blood from the cells into the veins M. The elasticity mentioned, united to the bridles which restrain over-distension, seem to occasion the smallest given quantity of blood to produce the intended distension. This distension occasions the diminution of the urethral capacity, presents a direct course for the emitting fluid, and increases the impetus of the semen.

Mankind at large unfortunately believe, that involuntary erection is not only a test of vigor, but that this consequently entitles them to indulgence in coitus. Nay, many expect they do themselves good thereby. The very reverse is the fact; involuntary effusion causing erection is a state of debility, and indulgence aggravates the malady. We cannot suppose that in articulo mortis there is a state of vigor, yet erection often takes place by the debility of the arteria penis; in fact, all involuntary phenomena are disease. I knew an instance of this erection in articulo mortis in the case of a catholic priest, of singular temperance; and very chaste persons often experience a contrary effect to that of priapism; there are none, however, more liable to fruitful coitus. Erection is an organic affection, often from a perverted intellect connected with a sterile seminiferous system. Persons laboring under the complaint of priapism, ought to apply cold water to the organ, abstain from excess, and resort to a tonic regimen, regulate the passions, &c. It is astonishing how soon some people recover from this disease by this means; man is the only animal that uses coitus where nature does not require it, or rather, I should say, forbids this function when

* In experiments on the ape species, and feeble salacious human subjects, I found that the arteries effused the injection with great facility into the cells. The injection I use shall be given in your next Journal if possible.

conception conception is effected. The Mahometans affront Christians in those points; and indeed they are so chaste, that the mind receives no excitement through the medium of the generative organs. Females who indulge in these gratifications are subject to abortion; and men to early sterility and palsy.

I find many complaining of what they name rheumatism, assignable wholly to this practice; and the practitioner easily discriminates this, by the pain being dull, and rather a want of power in the member suffering. I have many instances of patients completely recovering by observing temperance.*

For a great number of years I have demonstrated an enlargement of the spermatic cord by fatty deposition, which gradually dilates the ring, and predisposes to hernia; this is usually the attendant of a lascivious disposition, and is often accompanied by herniæ. I know that a learned and liberal world will forgive me, in offering a hint on herniæ which struck me on occasion of my being called in to a very obstinate scrotal case. The rupture was an old case; many however die by protracted stricture even in old age. How would it answer in such an instance, to make a bold incision, extirpate the testicle, return the intestine, and excite accretion of the scrotum? or in young men return the testicle (which would perform its office perfectly well) with the gut into the abdominal cavity, and by inflammation obliterate the scrotal cavity? Adhesion of the ring does not seem so invulnerable as this plan, where there is no exit for the gut.

THE FEMALE SEX.

Mankind may, in extenuation of their folly, plead a number of apologies for their misconduct; but, surely, the human species, especially woman, experiences no organic furor naturally. The connexion of the sexes in human nature seems rather an intellectual passion, in *temperate climates in particular*, than an animal appetite, till perversion inverts the laws of God. In man, the sexes are attracted from sentiment, not from sensation of organic principle; hence I find some savage nations very indifferent to the sex, as well as idiots. Furor uterinus is not so unfrequent, even in Europe, as some imagine. In ardent climates, where idleness,

* Nature is the work of a *wise God*—every agent is formed for and adapted to efficiency, and every organic exertion which oversteps these sacred limits, perverts and deranges organic structure, induces debility, precludes those principles on which health and pleasure depend, and promotes those convulsive actions which give rise to pain.—Vide Dr. Ramsay's first Fasciculus of the Anatomy of the Heart and Brain, *note*, p. 6 and 7.

romantic romantic fancy, luxury, and want of principle, unhinge all the laws of nature and religion, this direful evil is frequent: I have often traced it from early and persevering licentiousness; the male becomes sterile, yet continues lascivious, the error originated always with the male. The nymphæ and clitoris of the female are constructed similar to the penis, and are highly vascular; the vessels become relaxed, are morbidly excited, predisposing them to be thrown into a similar state as those of the male in priapism. The idea of intellectual passion has now, *if any*, a very feeble share in the excitement; appetite becomes the primary cause, as in the brute; the case becomes very obstinate: old men, connected with young women, often occasion this lamentable evil. I firmly believe, that, in both cases, temperance being observed, joined to a tonic regimen, conception would put a period to furor.*

Practitioners in warm climates, by a little attention to the symptoms I have mentioned, (to the pain in the cerebellic region, megrim, pained eyes, their tension, morbid phantoms of various colors, clouds, &c. false rheumatism, &c. in the female as well as the male,) may put a period to lamentable individual and family calamities.

*No.* 3, *Villa Place*, ALEX. RAMSAY.†

*Walworth Common, London,*

*Feb.* 10, 1814.

---

*For the Medical and Physical Journal.*

*A Case of Fracture, attended with Symptoms of unusual Violence, relieved by an extensive longitudinal Incision through the Fascia of the Limb; by* Mr. Mackesy, of Palermo.

THE severity of symptoms, and formidable appearance which marked the following case of simple fracture, together with the mode of treatment found effectual in their relief,

---

* It may be proper to inform the reader, that my giving lectures on the animal economy, not only in America but Europe, to select companies of gentlemen, brought me extensively acquainted with the facts stated. In temperate climates, among the Portuguese, British, &c. priapism seemed more prevalent than furor: in ardent climates, furor came more frequently before me than priapism.

† The author is engaged in an extensive work upon the Anatomy and Physiology of the Human Body; in the third fasciculus of which, the preceding subject will be more fully explained. In the mean time, a favorable opportunity is offered for discussing the hints which are now suggested, and which are supported by considerable experience.

## Figures and Tables

**Figure f1:**